# The crystal structure of the activin B:Fst288 complex and computational insights into the broad antagonistic activity and specificity of follistatin

**DOI:** 10.1016/j.jbc.2026.113459

**Published:** 2026-08-17

**Authors:** Lucija Hok, Ryan G. Walker, James A. Howard, Melissa M. Gouge, Eleanor M. Mast, Chandramohan Kattamuri, Erich J. Goebel, Thomas B. Thompson

**Affiliations:** 1 Department of Molecular & Cellular Biosciences, College of Medicine, University of Cincinnati, Cincinnati, Ohio, USA; 2 Department of Pharmacology & Systems Physiology, College of Medicine, University of Cincinnati, Cincinnati, Ohio, USA

**Keywords:** activin B (ActB), follistatin 288 (Fst288), transforming growth factor beta family (TGF-β), X-ray crystallography, molecular dynamics simulations (MDS)

## Abstract

Members of the transforming growth factor-β (TGF-β) family regulate essential biological processes, and their activity is tightly controlled by extracellular antagonists like follistatin 288 (Fst288). While Fst288 potently inhibits several ligands, including activins A and B, GDF8, and GDF11, the structural basis for its interaction with activin B (ActB) has remained largely uncharacterized. This lack of data has limited our understanding of how Fst288 achieves such broad inhibitory activity while maintaining ligand-specific selectivity. In this study, we resolved the crystal structure of the ActB:Fst288 complex at 2.7 Å resolution. Our findings reveal that while Fst288 utilizes a conserved receptor-blocking mechanism, ActB engages the antagonist through a modified structural mode. Most notably, the fingertips of ActB form direct, unique contacts with the third follistatin domain (FSD3). This interaction disrupts the intermolecular head-to-tail cooperativity typically seen in Fst288 dimers, shifting the stabilization of the complex toward individual ligand-domain affinities. Computational analysis supports a model where ActB relies more on direct contacts with Fst288, whereas ActA relies on the intermolecular head-to-tail Fst288 interactions. Further computational analysis indicates ActB is more flexible than ActA and GDF8. These results suggest that Fst288’s broad potency arises from a dynamic interplay between ligand flexibility and antagonist conformational plasticity that can accommodate different ligand surfaces. By elucidating unique characteristics of the ActB:Fst288 interface, this study deepens the understanding of ligand selectivity and provides a framework for the rational design of targeted TGF-β antagonists.

The transforming growth factor-β (TGF-β) family of cytokines regulates numerous physiological processes, including cell proliferation, differentiation, and tissue homeostasis. Members of this family are broadly categorized into 3 subgroups: TGF-βs, activins/inhibins, and bone morphogenetic proteins (BMPs). TGF-β ligands are typically disulfide-linked dimers with a characteristic cysteine-knot fold. They signal through the canonical pathway by forming ternary complexes with 2 type II and 2 type I serine/threonine kinase receptors. Upon ligand binding, type II receptors phosphorylate and activate type I receptors, which in turn, phosphorylate intracellular Smad proteins. Activated Smad proteins accumulate in the nucleus and function as transcription factors to regulate gene expression (1).

The actions of TGF-β ligands are tightly regulated by extracellular antagonists with diverse specificity profiles: some inhibit a narrow subset of ligands, while others exhibit broader activity and consequently, exert differential biological functions. One such broad spectrum antagonist is follistatin 288 (Fst288), a multidomain glycoprotein composed of an N-terminal domain (ND) and 3 follistatin domains (FSD1–3), each containing EGF-like and Kazal protease inhibitor-like domains (Figs. 1*A* and S1). Together with Fst315 and Fstl3, Fst288 belongs to the group of Fst domain-containing antagonists that differ in their architecture, binding characteristics and specificity for ligands (2, 3). Fst315 is a splice variant that contains an additional acidic C-terminal tail, while Fstl3 has a domain structure similar to that of Fst288, but lacks FSD3.

**Figure 1:**
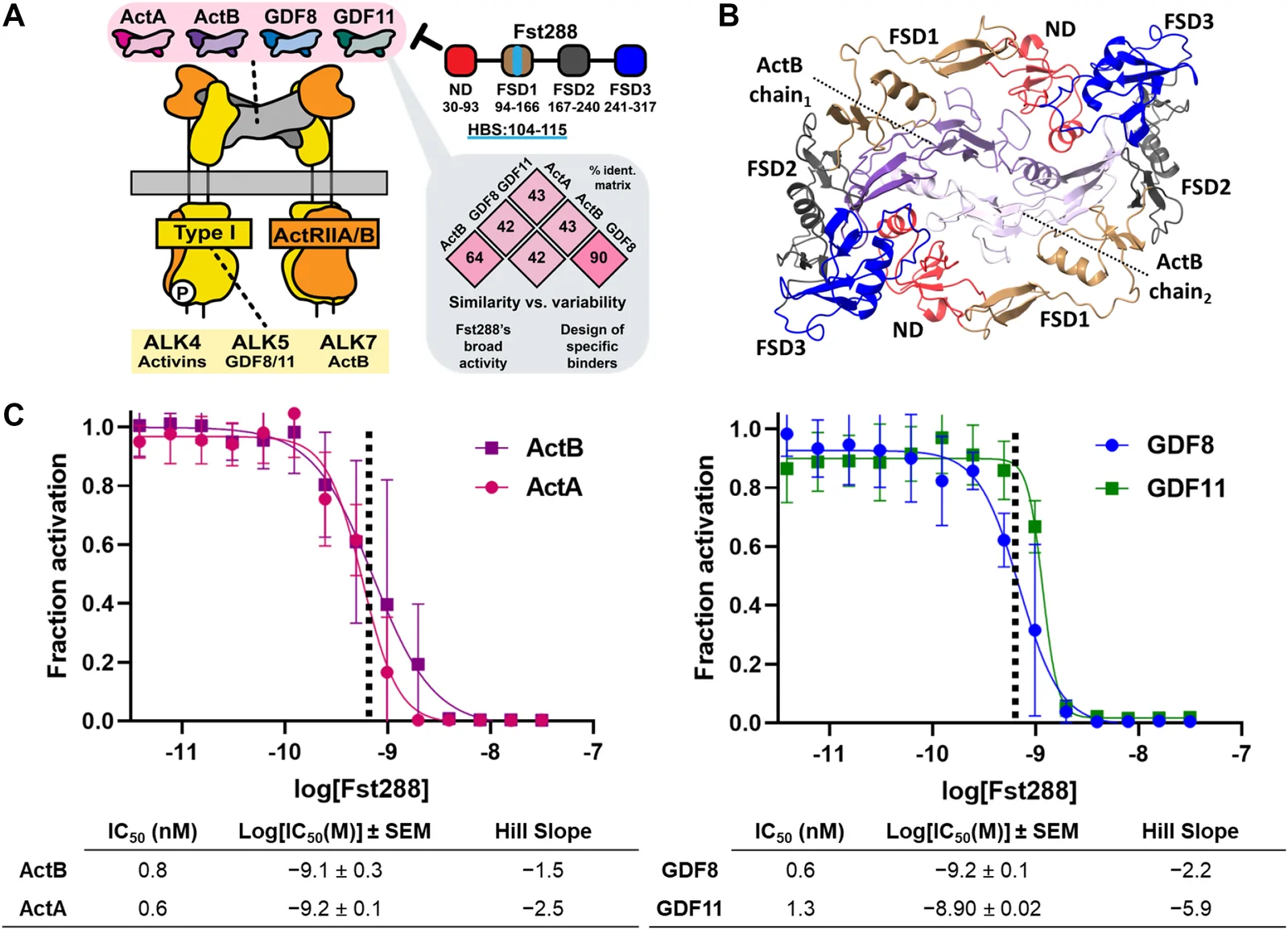
**Mechanism and Inhibitory Activity of Fst288 Toward Activins.***A*, schematic of the inhibition of activin signaling by Fst288 and sequence identity matrix (%) calculated for the growth factor domain. *B*, *top* view of the crystal structure of the ActB:Fst288 complex. *C*, luciferase reporter assays showing inhibitory activity following titration of Fst288 against a constant concentration (0.62 nM, *dashed line*) of activin *B* (*purple*) and activin *A* (*pink*) of the *left*, and GDF8 (*blue*), GDF11 (*green*) on the *right*, in HEK293 (CAGA)_12_ cells.

The widespread defects observed in the *Fst* knockout mice, including impaired breathing and developmental malformations in the whiskers, teeth, and muscles, underscore the broad antagonistic nature of Fst288 (4). Initially identified as a strong inhibitor of activin A (ActA), Fst288 has also been shown to inhibit activin B (ActB), GDF8, and GDF11 at nanomolar concentrations (5, 6). In contrast, it has minimal effects on BMP signaling and does not inhibit the TGF-β subgroup (7). Crystal structures of Fst-type molecules in complex with ActA, GDF8, or GDF11 reveal a common neutralization mechanism in which two Fst molecules occlude both receptor-binding sites of the ligand (5, 8, 9, 10, 11, 12).

Numerous studies have examined the contribution of individual Fst domains to activin antagonism using mutational and domain-swapping approaches (10, 13, 14, 15, 16, 17, 18, 19). Both the ND and FSD1–2 are required for effective ActA inhibition (13), whereas an ND-FSD1-FSD1 construct selectively antagonizes GDF8 (14, 16, 19). Point mutations in the ND and FSD2 are equally detrimental to antagonism, suggesting that affinity for ligands is spread out over both domains (14, 18). Although FSD3 does not directly contact the ligand, it enhances binding *via* cooperativity by interacting with the ND of the adjacent Fst288 molecule in a head-to-tail configuration. Addition of FSD3 can rescue mutated forms of Fstl3 and Fst288 with reduced antagonism (10, 13, 14). The lack of cooperativity in Fstl3 and ΔFSD3 constructs lead to the speculation that the ND–FSD3 interaction underlies Fst288's broad activity (14). Nevertheless, differences in antagonistic potency toward distinct TGF-β ligands suggest that ligand-specific structural features also play a critical role in determining ligand selectivity.

While ActA, GDF8, and GDF11 are structurally well characterized, structural insight into ActB and its interactions with antagonists remains limited (5, 8, 9, 10, 11, 12, 15, 20, 21, 22, 23, 24, 25, 26). To address this gap, we determined the crystal structure of ActB in complex with Fst288 (PDB ID: 9Z3T). Comparison with previously resolved activin:Fst structures revealed that ActB engages Fst288 distinctly at the type I receptor binding site, relying more on its fingers than other activins. Moreover, ActB directly interacts with FSD3 through its highly acidic fingertips, disrupting the ND–FSD3 interaction and hindering cooperativity. These findings support a model in which Fst288 adapts its binding mode to ligand-specific structural features. The structural characterization of the last remaining high-affinity activin:Fst288 complex provides new insights into the regulation of TGF-β signaling by endogenous antagonists, and offers a framework for the rational design of selective antagonists (27, 28, 29, 30, 31) (Fig. 1*A*).

## Results

### The structure of the ActB:Fst288 complex

The structure of ActB bound to two Fst288 molecules was resolved using X-ray crystallography to 2.7 Å resolution (Table 1), representing the first structure of ActB in complex with a known antagonist. As observed in previously published activin:Fst structures (5, 8, 9, 10, 11, 12), 2 molecules of Fst288 encircle the dimeric ligand occluding both type I and type II receptor binding sites (Fig. 1*B*). The ND domain occupies type I receptor site, contacting the concave surface of the ligand fingers and the wrist helix of the opposite chain_2_, while FSD2 and, to a lesser extent, FSD1 bind to the convex surface of the fingers, overlapping with the type II binding epitope.

**Table 1:** Data Collection and Refinements statistics (Molecular Replacement)

Crystallographic Parameter	Native^a^
Data collection	
Space group	C 1 2 1
Unit cell dimension (Å, °)	*a* = 157.3, *b* = 77.1, *c* = 108.8
	α = γ = 90, β = 132.8
Resolution (Å)	42.7−2.7 (2.8−2.7)
*R*_merge_	0.044 (0.749)
*R*_pim_	0.032 (0.549)
Mean (*I*/σ*I*)^b^	14.2 (1.2)
CC_1/2_	0.998 (0.636)
Completeness (%)	94.05 (87.52)
Multiplicity	2.7
Wilson plot B factor (Å^2^)	83
Model refinement	
Total reflections	64,592 (6314)
Unique reflections	24,284 (2496)
R_work_/R_free_ (%)^c^	22.8/27.5
Number of atoms (molecules)	
non-hydrogen atoms	6169
macromolecules	6141
ligands	28
solvent	0
Protein residues	806
Average B-factor (Å^2^)	113
RMS deviations	
Bonds (Å)	0.003
Angles (°)	0.58
Ramachandran plot	
Favored (%)	94.61
Allowed (%)	5.39
Outliers (%)	0
Rotamer outliers (%)	1.01
Clashscore^d^	6.31

a Values in parentheses are for highest resolution shell (2.8–2.7).

b Mean (*I*/σ*I*) is defined as < merged < *I*h >/sd (< *I*h >)>≈ signal/noise.

c *R*_free_ calculated from 5% of initial total number of reflections.

d Determined by MolProbity.

### Fst288 potently inhibits activin ligands

Since Fst288 adopts a similar receptor-blocking binding mechanism across activin ligands, we next assessed whether its inhibitory potency differs among ActB, ActA, GDF8, and GDF11. Using a HEK293 (CAGA)_12_ luciferase reporter assay, Fst288 was titrated against a constant concentration of each ligand. Fst288 inhibited activin class ligands with comparable potency at nanomolar concentrations (Fig. 1*C*) and failed to antagonize BMP2 (Fig. S2).

### Overall features of the ActB:Fst288 interaction

**Figure 2:**
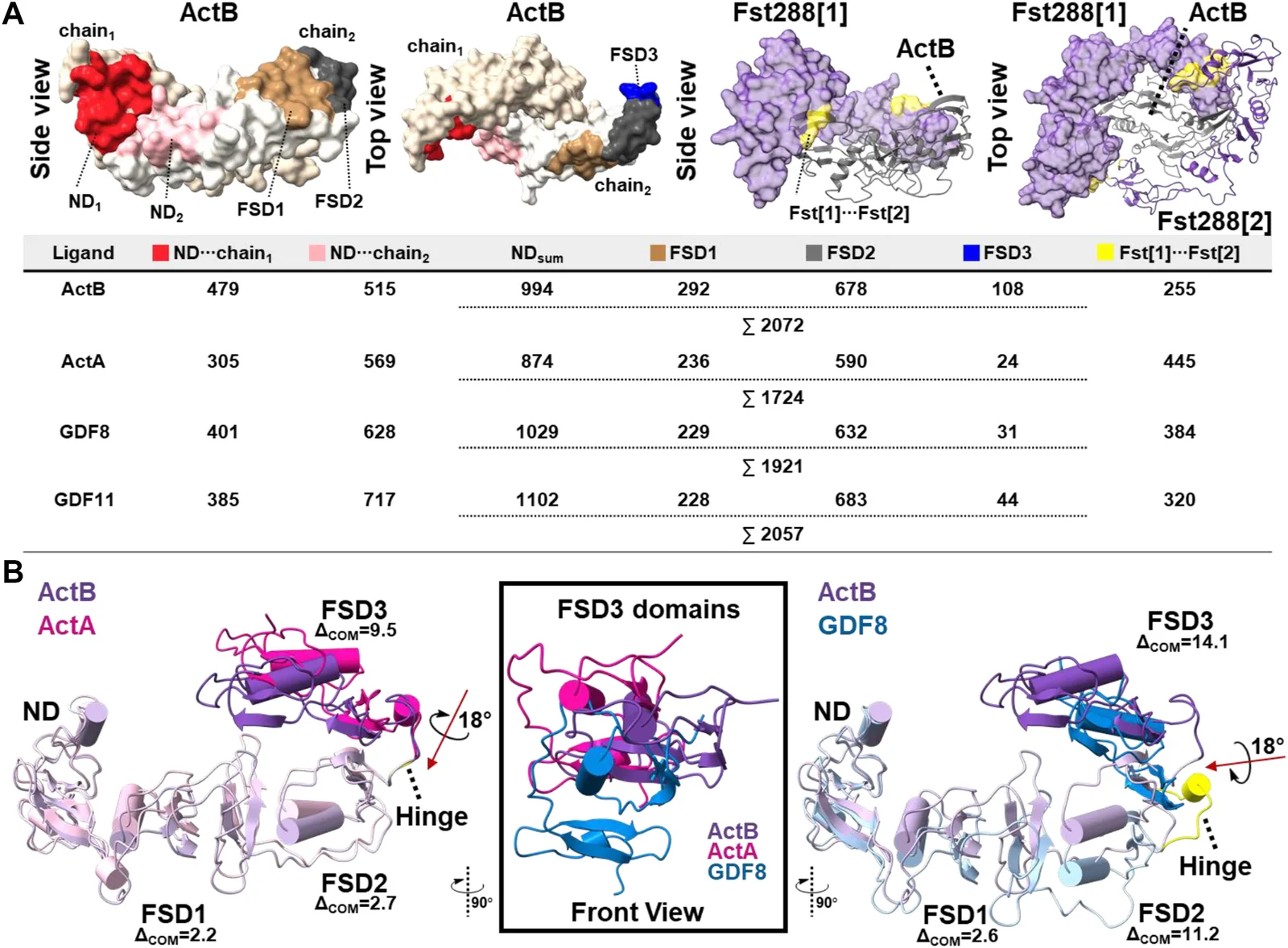
**Structural Comparison of Activin:Fst288 Complexes.***A*, buried surface area (in Å^2^) between ActB and individual Fst domains: ND with chain_1_ in *red*, ND with chain_2_ in *pink*, FSD1 in *brown*, FSD2 in *grey*, FSD3 in *blue*; and between the two Fst in *yellow*. Calculated values (BSA ≥ 15 Å^2^) for activins are given in the table below. *B*, dynamics of Fst288 in the ActB complex compared to ActA (*left*) and GDF8 (*right*), aligned through the ND. *Red arrows* indicate hinge axis and Δ_COM_ values (in Å) represent the displacement of the centers of mass of individual domains in ActA and GDF8 relative to ActB. Center: front views of the FSD3 domains from ActB, ActA, and GDF8 (*left* and *right* images rotated vertically by 90°).

ActB consists of 2 inhibin β_B_ chains whose growth factor domains share 64% sequence identity with the β_A_ chains of ActA (32), whereas GDF8 and GDF11, which are 90% identical, share only ∼40% identity to the inhibin β chains (12) (Fig. 1*A*). Despite its greater similarity to ActA, the Fst288:ActB interface more closely resembles that of GDF8 and GDF11, forming more extensive contacts and burying an additional 348 Å^2^ of surface area (Fig. 2*A*). Notably, the distribution of ND interactions differs: whereas in other complexes the ND primarily engages the prehelix loop and wrist helix of chain_2_, in ActB the ND engages both chains equally, with the largest contact area formed between the ND helix and the concave side of chain_1_ (Fig. 2*A*). Even more strikingly, unlike in other activin complexes where FSD3 makes little to no contact with the ligand, FSD3 in the ActB complex directly engages the ligand fingertips. The type II receptor binding epitope is largely conserved across all complexes with similar contact areas, despite FSD1 in ActA complex being vertically displaced downward toward fingers 1 and 2 (Fig. S3).

### The role of ligand and Fst288 flexibility in forming high-affinity complexes

Crystal structures of activin ligands resolved to date have revealed substantial variability in interchain conformations of the growth factors, arising from the wrist helix being only loosely tethered to the fingers of the opposite monomer (5, 8, 9, 10, 11, 12, 15, 20, 21, 22, 23, 24, 25). How this flexibility influences interactions with binding partners, however, remains unknown. In the ActB:Fst288 complex, the ligand adopts a conformation similar to GDF8 and GDF11, with an interchain angle of 111°, whereas ActA adopts a ∼10° more closed conformation, leading to a vertical shift of FSD1 toward fingers 1 and 2 (*vide supra*). Despite these differences, variations in fingertip curvature result in complexes of comparable length and overall architecture.

To investigate ligand flexibility, we performed molecular dynamics (MD) simulations on both the Fst288-bound complexes and their apo forms — the later generated by removing the Fst288 molecules in the corresponding crystal structures. Simulations of apo ActA and GDF8 showed slightly greater flexibility relative to their bound states, but retained their interchain conformations, indicating that these geometries represent stable local minima (Fig. S4). In contrast, apo ActB exhibited pronounced global rearrangements, with substantial root mean square deviation (RMSD) fluctuations, and a transition from the closed state seen in the Fst288 complex (∼110°) to a more open state (∼130°), approaching that seen in the ActB:BMPR2 crystal structure (142°) (22). Although Fst288 binding stabilizes ActB, the prehelix and wrist helix region remains the most dynamic among the ligands, as evidenced by the largest occupied volume during MD simulations (Fig. S4). This flexibility shifts the ND domain of Fst288 closer to the more stable ActB fingers, resulting in an increased contact area with chain_1_.

To further probe the contributions of this region, we computationally exchanged the prehelix and wrist helix regions between ActA and ActB. The ActA^B^ (ActA with prehelix and wrist of ActB) chimera displayed increased flexibility, whereas ActB^A^ was more stable than the corresponding wild-type forms (Fig. S5). Notably, the ActA^B^ mutant did not transition to an open conformation, indicating that the interactions between the wrist helix and the opposing chain fingers also contributes to the overall geometry of the ligand.

Fst288 also displays ligand-dependent variability in its domain positioning. Alignment of ND from the ActB-, ActA-, and GDF8-bound Fst288 structures shows that small interdomain rearrangements accumulate into a pronounced repositioning of FSD3 (Fig. 2*B*). Whereas FSD3 contacts ND of the adjacent Fst288 molecule in other complexes, in ActB it shifts closer to the ligand fingers. This difference was even more pronounced in MD simulations where FSD3 in the ActB:Fst288 complex moves even closer to the ligand fingers, stabilizing ionic interactions between ActB fingertip 2 and positively charged residues on FSD3 (Fig. S6). These observations indicate that Fst288 adopts alternative conformations to accommodate and stabilize distinct activin ligands.

Although a structure of Fst288 in its ligand-free form is not yet available, we were interested in the orientation of its domains in the absence of a ligand. We removed GDF8 from the complex and performed MD simulations. Both Fst288 monomers and dimers exhibited substantial domain movements without ligands. These structural dynamics were primarily driven by ionic interactions – intramolecular in the monomer and intermolecular in the dimer – between positively charged residues on FSD1 and negative on FSD3 (Fig. S7). This indicates that Fst288 possesses intrinsic conformational plasticity that enables Fst288 to optimize contacts across a range of ligands.

### ND domain interacts more extensively with ActB chain_1_ compared to other activin ligands

**Figure 3:**
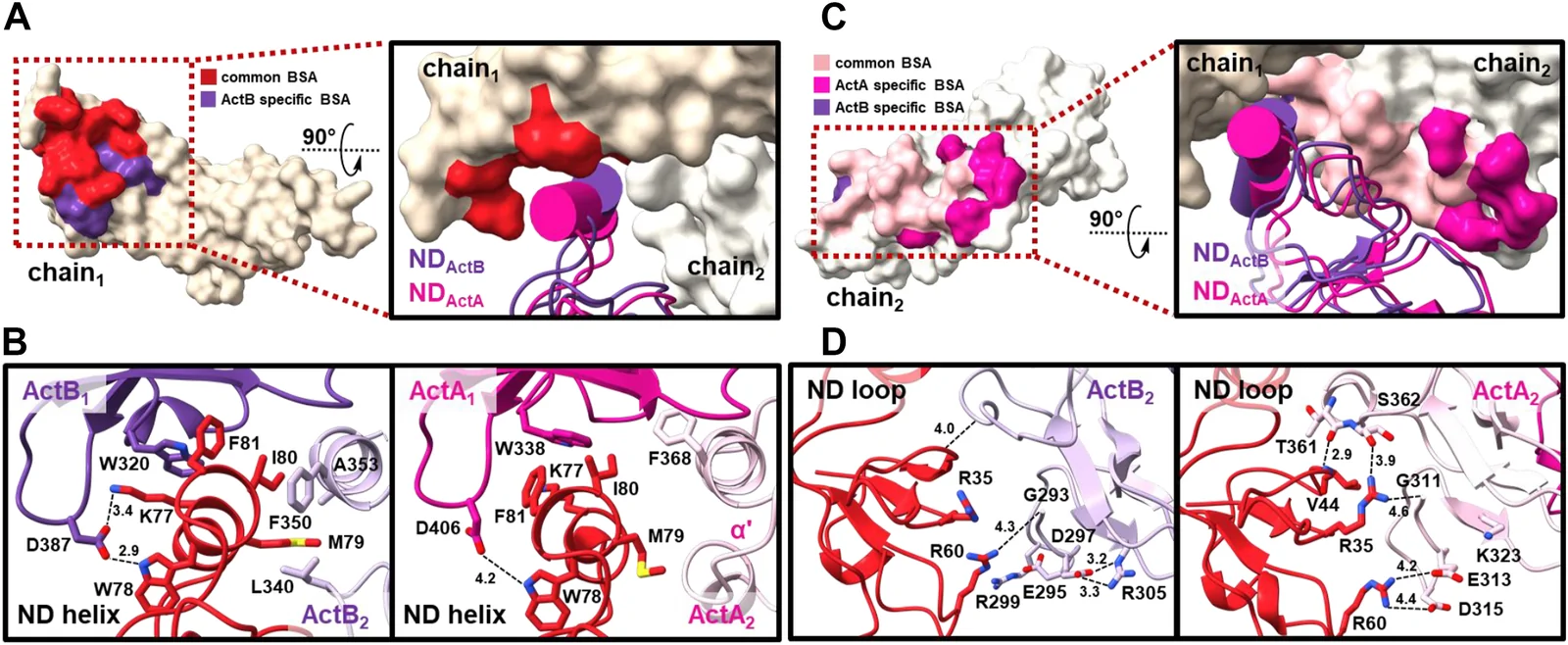
**Type I Ligand Interface with the ND Domain.***A*, the buried surface area on chain_1_ by the ND that is shared between ActB and ActA is colored *red*, while the area specific to ActB is shown in *purple*. On the *left*, only the ActB chain_1_ is shown in beige surface representation. On the *right*, both chains and the ND helices are shown from a view rotated 90° horizontally. *B*, detailed view of the ND helix binding site within the concave region of ActB (*left*) and ActA (*right*). *C*, the buried surface area on chain_2_ by the ND that is shared between ActB and ActA is colored *pink*, while the area specific to ActA is shown in magenta and to ActB in *purple*. On the *left*, only the ActA chain_2_ is shown in beige surface representation. On the *right*, both chains and the ND are shown from a view rotated 90° horizontally. *D*, detailed view of the interaction between the ND loops and the N-terminus and wrist helix of ActB (*left*) and ActA (*right*).

Comparison with previously reported Fst288 and Fstl3 structures highlights a malleable ND-ligand interface. In the ActB:Fst288 complex the ND helix is positioned 2.3 Å deeper within the type I site of ActB than in ActA, resulting in more extensive contacts with chain_1_ (Fig. 3*A*). This deeper positioning is enabled by the absence of steric clashes with the prehelix region on chain_2_ which is disordered in ActB, but forms an α′ helix in ActA (Fig. 3*B*). Consistent with this, ND positioning in GDF8 and GDF11 complexes more closely resembles that of in ActB, likely due to their shorter prehelix loops (Fig. S8*A*). Consequently, ND helix in ActB, GDF8 and GDF11 complexes forms numerous hydrophobic interactions with disordered prehelix loop and wrist helix that are not formed in ActA (Fig. 3*B*). Similar interactions have been observed for an α helix in type I receptors at the same position, suggesting a mimicking approach of Fst to outcompete receptors (25, 26). At the ligand fingertips, by contrast, interactions with ND helix become more ionic in nature (Figs. 3*B* and S8*A*), adapting to those observed in type I receptors (25, 26). Other antagonists employ similar types of interactions to outcompete receptors (5, 10, 33, 34).

Differences are also observed in how ND interacts with prehelix loop/wrist helix of chain_2_. In general, fewer contacts are formed between ND and chain_2_ of ActB compared to other activins (Figs. 3, *C*, *D*, and S8*B*). To help determine how important the prehelix region is to the position of ND, we examined structures from MD simulations of the chimeric ligands ActB^A^ and ActA^B^. Here, we observed that in the ActB^A^:Fst288 complex, the α′ helix was formed as in wild type ActA, resulting in a 3.5 Å shift of the ND helix away from its position in the ActB:Fst288 crystal structure (Fig. S9). Interestingly, in the ActA^B^ mutant, while the prehelix region remained disordered, we did not notice the expected movement of ND helix closer to the concave interface. Instead, only a slight shift at the base of ND helix was detected, suggesting that, beyond steric hindrance, interactions with ligand fingers and fingertips also play an important role in ND positioning.

### Type II ligand epitope comprises a hydrophobic core and a polar periphery

**Figure 4:**
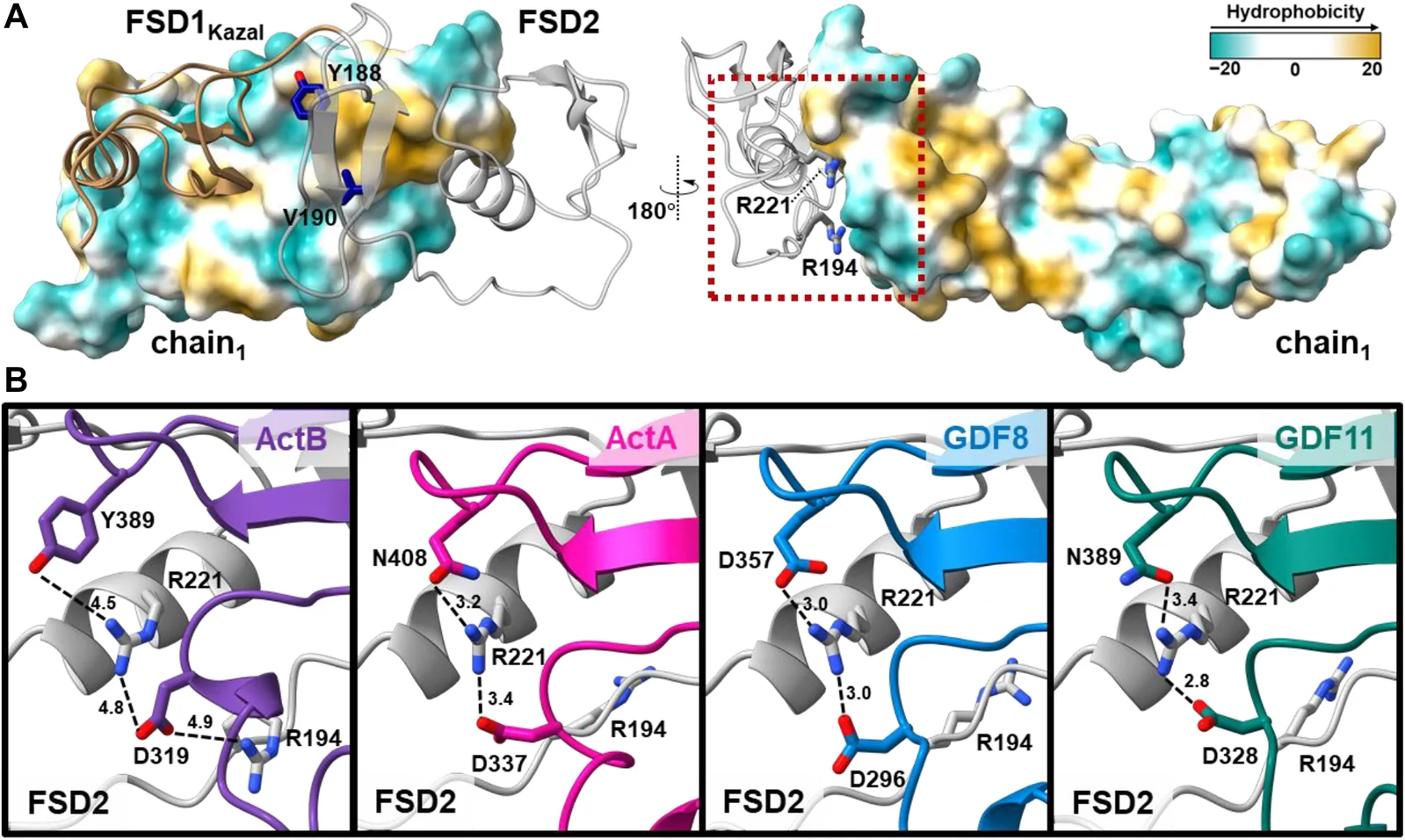
**Type II Ligand Interface with the FSD1 and FSD2 Domains.***A*, *Left*: Surface representation of ActB chain_1_ colored according to hydrophobicity, showing hydrophobic contacts between the FSD1-FSD2 domains and the central patch on the knuckle epitope. *Right*: *Front* view of the FSD2 interaction with ActB fingers. The boxed region is shown enlarged in *panel B*. *B*, detailed views of the interactions between R221 (and R194) in FSD2 and the ligand fingertips.

The majority of the FSD1/FSD2 binding interface is composed of numerous conserved, non-polar residues. These residues form consistent interactions across all 4 complexes and similarly engage the hydrophobic “hotspot” utilized by type II receptors (22, 25, 26). For example, a conserved leucine on finger three of the ligands interacts with Y188 in FSD2. This mirrors typical interactions, as Y188 structurally aligns with Y67 in BMPR2, F61 in ActRIIA, and Y60 in ActRIIB. Furthermore, an isoleucine—part of the conserved IAP motif in activins and BMPs—interacts with V190 (Fig. 4*A*).

In addition to the hydrophobic interactions, a conserved ionic interaction is formed between an aspartate on fingertip 1 and R221 on FSD2. R221 is further stabilized by hydrogen bond-accepting residues on fingertip 2: Tyr in ActB and Asp or Asn in other activins (Fig. 4*B*). In ActB complex, the conserved Asp also participates in a unique salt bridge with R194 on a loop in FSD2. This positions the FSD2 loop closer to the ligand as compared to other activins (Δ_ActA_ = 5.5 Å, Δ_GDF8_ = 4.3 Å, Δ_GDF11_ = 3.4 Å). Notably, these ionic contacts with FSD2 do not have direct counterparts in type II receptors, suggesting an important role beyond competition with receptors for binding, such as in determining selectivity.

### FSD3 interacts with ActB fingertips at the expense of intermolecular Fst288 contacts

**Figure 5:**
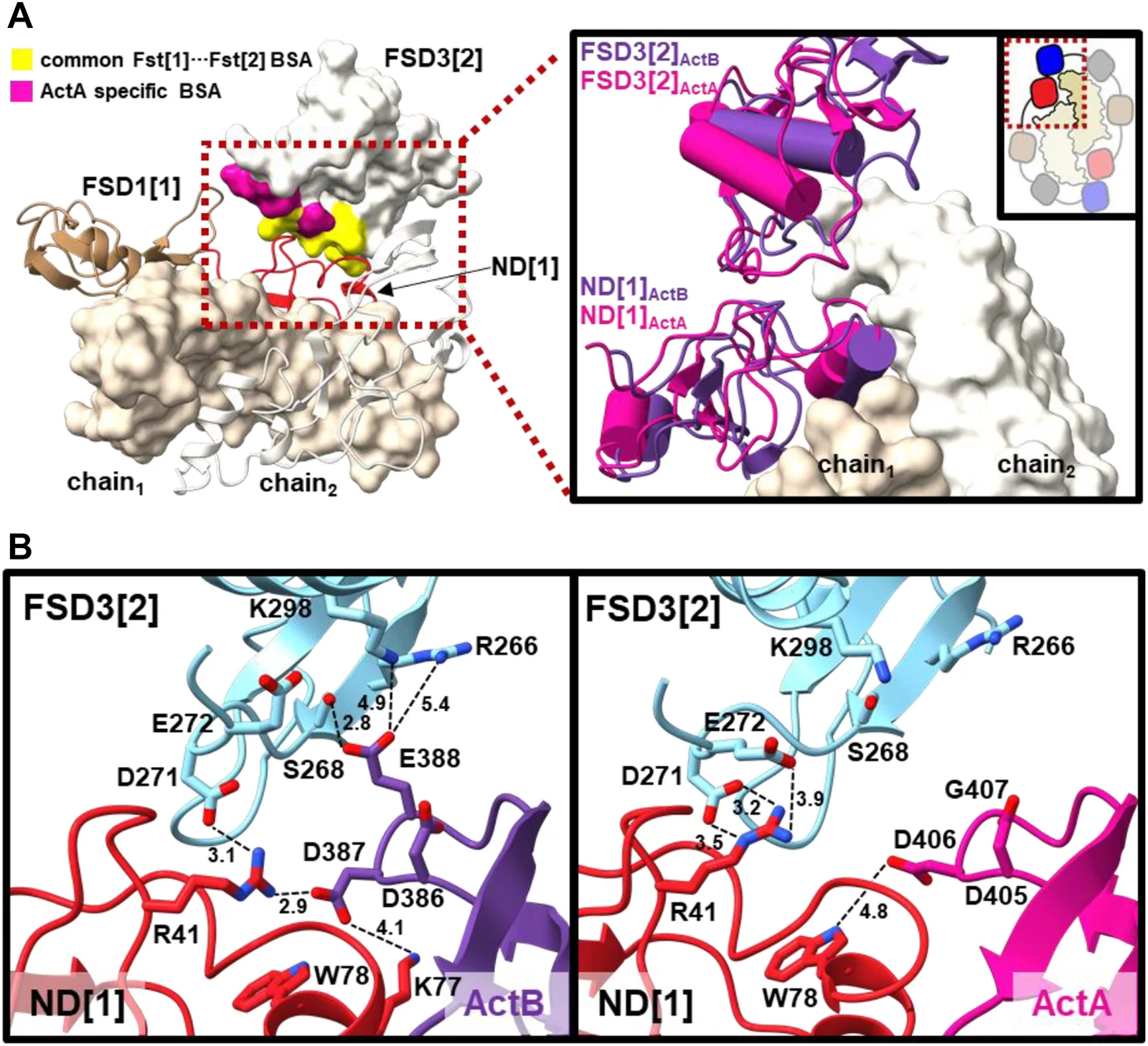
**Interface between Ligands and Two Fst288 Molecules.***A*, on the *left*, the buried surface area on FSD3[2] by the Fst[1] that is shared between ActB and ActA is colored *yellow*, while the area specific to ActA is shown in *magenta*. ActA chain_1_ is shown in beige surface representation, chain_2_ in *white* cartoon, ND[1] in *red*, and FSD1[1] in *brown*. On the *right*, the ND and FSD3 domains from the ActB and ActA complexes are shown from a view indicated in the *top right* corner. *B*, detailed view of the interactions between the FSD3[2] (*light blue*), ND[1] (*red*) and ligand in the ActB (*left*, in *purple*) and ActA (*right*, in *pink*) complexes.

In the ActB:Fst288 complex, FSD3 directly engages with the ligand fingertips (Fig. 5*A*), an interaction not observed in other ligand:Fst288 complexes. These contacts are mediated by E388 in ActB, which forms hydrogen bonds with residues in FSD3 (Fig. 5*B*). In ActA, this position is occupied by glycine, and a lysine in GDF8 and GDF11, neither of which supports comparable interactions (Figs. 5*B* and S8*C*). As a result, FSD3 is positioned closer to the ActB ligand than in the other complexes, leading to a concomitant reduction in contacts between FSD3 and the ND of the adjacent Fst288 molecule.

### Computational studies support less dependence on cooperativity in the ActB:Fst288 complex

**Figure 6:**
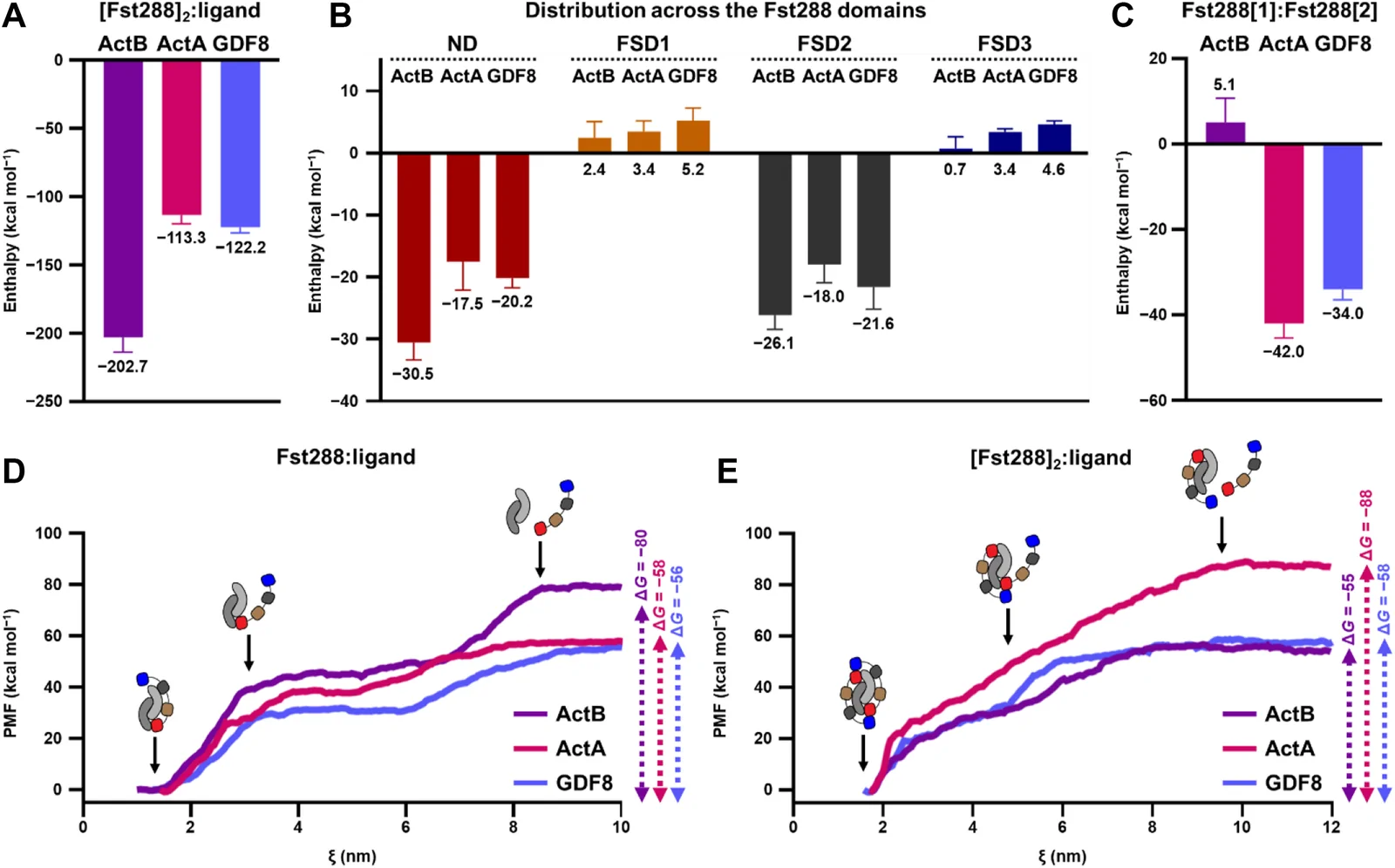
**Computational Analysis of Interaction Enthalpy and Gibbs Free Energy in Activin:Fst288 Complexes.***A*, calculated MM-GBSA enthalpy between two Fst288 molecules and ligands (in kcal mol^−1^). Values represent averages from 5 MD replicas. *B*, distribution of calculated MM-GBSA enthalpy across individual Fst288 domains (in kcal mol^−1^). Values represent averages of both domains from 5 MD replicas. *C*, calculated MM-GBSA enthalpy between the two Fst288 molecules (in kcal mol^−1^). Values represent averages from 5 MD replicas. Potential of mean force curves along the reaction coordinate, calculated from umbrella sampling simulations for dissociation of the first Fst288 from ligands. *D*, or the second Fst288 from Fst288:ligand complexes. *E*, with corresponding Δ*G* values indicated on *right* (in kcal mol^−1^).

Structures of ligand:Fst288 complexes clearly show accommodations in both the ligand and antagonist that occur to maintain high-affinity interactions. Given the wealth of structural information, we wanted to evaluate these differences using computational methods. For this we employed MD simulations combined with MM-GBSA calculations to determine enthalpy of interactions (Table S1). The estimated interaction enthalpy of the ActB:Fst288 complex is lower than that of the corresponding ActA and GDF8 complexes (Fig. 6*A*). When total interaction enthalpy with the ligands is decomposed by individual Fst288 domains, calculations indicate that the ND and to a lesser extend FSD2, contribute more strongly to ActB binding than to ActA or GDF8 (Fig. 6*B*). FSD1 consistently contributes unfavorably to binding across all 3 studied systems, as does FSD3 in the ActA and GDF8 complexes, but not in ActB.

Interestingly, the interaction enthalpy between the two Fst288 molecules is quite favorable in the ActA and GDF8 complexes, but slightly unfavorable in the ActB complex (Fig. 6*C*). These results suggest that intermolecular Fst288-Fst288 interactions contribute to stabilization of the ActA and GDF8 complexes, whereas interaction with ActB relies more on the individual affinities of each Fst288 molecule. This is consistent with previous studies that, based on the slope of luciferase reporter assay curves, suggested that cooperativity is prevalent in ActA and GDF8 antagonism (14). Our luciferase assays support this as well: while higher negative hill slope values in ActA and GDF8 indicate cooperativity, the value of −1.5 for ActB suggests a lack of cooperativity (Fig. 1*C*).

To further computationally investigate the contribution of cooperativity, we used umbrella sampling to calculate free energy profiles for the dissociation of both Fst288 molecules. The calculated Gibbs free energy for the binding of the first Fst288 molecule is lower for ActB than for ActA and GDF8 (−80 kcal mol^−1^ vs. −58 kcal mol^−1^, and −56 kcal mol^−1^, respectively) (Figs. 6*D*, S10 and S11). All 3 energy profiles exhibit biphasic behavior with an initial increase for the dissociation of FSD1−3 and a second increase corresponding to dissociation of the ND. The length of the plateau and the final energy level are notably larger in ActB, suggesting that ND binds most strongly to ActB. A similar pattern was observed in dissociation profiles of the ND alone (Fig. S12).

The calculated Gibbs free energy for binding of the second Fst288 is lowest for ActA (−88 kcal mol^−1^), followed by GDF8 (−58 kcal mol^−1^), and ActB (−55 kcal mol^−1^), further supporting the importance of the intermolecular Fst288-Fst288 interactions in the overall GDF8, and especially ActA affinity (Fig. 6*E*). When considering these additional interactions, the shape of curves changes and becomes monophasic, indicating resistance against disruption of the Fst288-Fst288 interaction. The summed Δ*G* of both binding events align with previous reports that Fst288 has higher affinity for ActA (−146 kcal mol^−1^) than for ActB and GDF8 (−135 kcal mol^−1^ and −114 kcal mol^−1^, respectively) (5, 6).

### The ActB:Fst288 complex exhibits similar behavior to ActA:Fst288 in heparin affinity experiments

**Figure 7:**
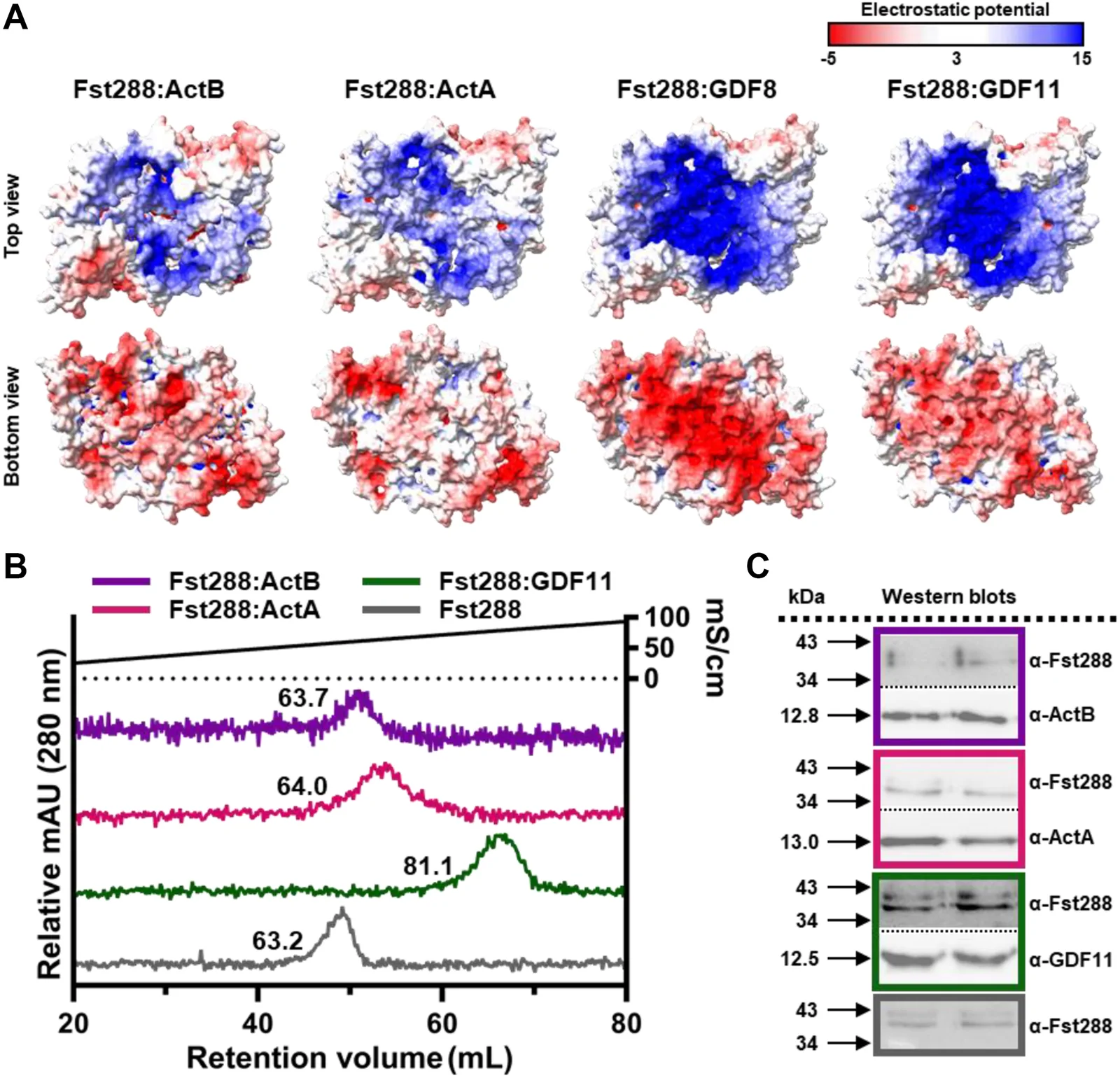
**Interaction of Activin:Fst288 Complexes with Heparin.***A*, *Top* and *bottom* views of the electrostatic potential surfaces for Fst288:ligand complexes, calculated using Adaptive Poisson-Boltzmann Solver (APBS), with positive regions in *blue* and negative in *red*. *B*, UV absorbance traces from heparin affinity chromatography for Fst288 alone (*gray*), and for Fst288 complexes with ActB (*purple*), ActA (*magenta*), and GDF11 (*green*). Proteins were eluted using a NaCl gradient. Conductivity values are indicated at each peak maximum. *C*, fractions from the heparin column for each sample (colored as in *panel B*) were analyzed by Western blot using corresponding primary antibodies.

Fst288 binds heparin and heparan sulfate (HS) through a heparin binding sequence (HBS) located on FSD1 (Fig. 1*A*) (35). Electrostatic surface potential maps of different ligand:Fst288 complexes are shown in Figure 7*A*. In the GDF8 and GDF11 complexes additional positively charged residues from the ligand contribute to create a continuous electropositive surface, which has been shown to significantly enhance heparin/HS binding compared with the ActA:Fst288 complex (11, 12). In contrast, similar to ActA, ActB lacks comparable positively charged residues (Fig. 7*A*).

To assess heparin affinity experimentally, purified ligand:Fst288 complexes were applied to heparin affinity resin and eluted with a NaCl gradient. Consistent with the electrostatic surface properties, ActB:Fst288 eluted at a NaCl concentration similar to that of the ActA:Fst288 complex, whereas GDF11:Fst288 complex required higher salt concentration for elution, indicating stronger interactions (Figs. 7, *B* and *C*).

## Discussion

Fst288 is a broad-spectrum, multidomain antagonist of TGF-β ligands, yet the molecular determinant underlying its ligand selectivity has remained poorly defined. By resolving the crystal structure of Fst288 in complex with ActB and integrating structural and computational analyses, we elucidate how Fst288 accommodates diverse ligands while preserving high inhibitory potency. These findings support a model in which intrinsic conformational plasticity of Fst288, together with ligand flexibility, enables adaptable binding modes across ligands, with Fst288-Fst288 interactions contributing in a ligand-dependent manner to complex stabilization.

From a global perspective, Fst288 binds activins in a similar fashion, surrounding them with 2 molecules. By utilizing the flexibility of its interdomain loops, Fst288 accommodates ligands whose interchain angles can range from 50° (PDB: 1NYS) to 142° (PDB: 7U5O), and constrains them all to a similar conformation with an interchain angle of ∼110°. Strong binding is achieved by mimicking structural features of receptors: *e.g.* the ND helix positions itself in the ligand's concave surface where the universal type I receptor motif would normally bind, while the EGF domain of FSD2 imitates the three-finger toxin fold of type II receptors on the knuckle epitope. In addition to these secondary-structure similarities, Fst288 also forms receptor-like interactions with the ligands, ensuring high affinity and effectively outcompeting receptor binding.

A defining feature of the ActB:Fst288 complex is the enhanced role of the ND in ligand binding. In ActB, the ND helix penetrates deeper into the type I receptor binding site, forming more extensive contacts with chain_1_ than observed in ActA. This positioning is enabled by the absence of a structured α′ helix in the ActB prehelix region, which imposes steric constraints in ActA. Computational studies indicate that, while dimers of ActA, GDF8 and ActB all appear flexible, there might exist different degrees of flexibility. MD simulations suggest that ActB is intrinsically more flexible than other activins and samples a wider range of interchain conformations in the absence of Fst288. Binding of Fst288 stabilizes a more closed ActB conformation, effectively shifting the ND into the dimer interface. These findings are consistent with a conformational selection mechanism in which Fst288 preferentially binds and stabilizes specific ligand conformers.

Flexibility also emerges as a key property of Fst288 itself. Comparison of multiple ligand-bound structures and MD simulations reveals that small interdomain rearrangements accumulate to produce substantial repositioning of FSD3. In ActA and GDF8 complexes, FSD3 stabilizes binding through head-to-tail interactions with the ND of the adjacent Fst288 molecule, giving rise to cooperative antagonism. In contrast, in the ActB complex, FSD3 is redirected toward the ligand fingertips, forming direct contacts mediated by acidic residues unique to ActB. This shift disrupts intermolecular Fst288-Fst288 interactions and eliminates cooperativity. Thus, cooperativity is not an intrinsic feature of Fst288 antagonism but arises when ligand geometry permits stabilization of the Fst288 dimer interface.

The effect of flexibility on overall affinity is best illustrated by the energy calculations. The more numerous contacts in the ActB:Fst288 complex, arising from stronger ND binding and direct interactions with FSD3, result in a more favorable interaction enthalpy compared to ActA and GDF8. However, the enthalpic gain from strong binding can be offset by a loss of entropy, as the interacting partners become more conformationally restricted. In other words, the tighter and more specific the interaction, the less entropically favorable it becomes. Considering higher flexibility of ActB in apo state shown in MD simulations, we speculate that enthalpy-entropy compensation might affect its affinity for Fst288. Namely, it is possible that restriction of ActB flexibility upon binding leads to a less favorable Gibbs free energy and lower affinity than measured for ActA (5, 6).

In a cellular context, interactions between ligands and antagonists may be further affected by binding to the extracellular matrix (ECM). Fst288 binds heparin and heparan sulfate (HS) and localizes to the cell surface, which facilitates endocytosis of Fst-bound ligands (35, 36). Previous studies have shown that the electrostatic properties of ligands alter the affinity of complexes for ECM. Accordingly, binding of GDF8 and GDF11 with localized patches of positive charge further increases the affinity of Fst for heparin and HS, whereas the ActA complex, which lacks such a composite positive surface, has an affinity comparable to that of Fst288 alone (11, 12). The ActB complex shows similar behavior to ActA. These interactions likely account for the nearly identical nanomolar IC_50_ values observed for all studied activins *in vitro*, despite differences in binding affinities. This suggests that both: interaction strength of complexes and ability to bind ECM, contribute to the high potency of Fst288.

The high adaptability of Fst288 and its reliance on other factors beyond direct ligand binding, such as cooperativity and localization to the ECM, raise questions regarding specificity for the activin subgroup. Comparison of aligned sequences from activins and non-binding TGF-βs reveals that the most striking differences occur at the ligand fingertips (Fig. S13). At fingertip 1, activins have an aspartate that interacts with R221 on FSD2, whereas TGF-βs carry a lysine. Similarly, the acidic fingertip 2 in activins stabilizes the ND helix, and additionally FSD3 in ActB, whereas in TGF-βs these residues are hydrophobic and surrounded by positively charged residues. This suggests that acidic fingertips are critical for antagonist recognition. Interestingly, the acidic nature of the fingertips within the activin class has also been shown to be important for type I receptor binding (26).

The most pronounced differences between BMPs and activins occur in the prehelix and wrist helix regions, which in BMPs form a rigid, high-affinity type I receptor-binding site, but remain highly flexible in activins (Fig. S13). This highlights the importance of ligand plasticity for strong Fst288 binding, which can be further enhanced through ligand-dependent cooperativity between 2 adjacent Fst288 molecules. An interesting case is represented by activin C and activin E, members of the activin subgroup that are insensitive to Fst288 antagonism despite their relative similarity to other activins (37, 38). Further biochemical and structural studies are required to determine whether unfavorable interactions or an inability to leverage cooperativity underlie their weak Fst288 binding.

In conclusion, this study demonstrates that specificity and potency of Fst288 arise from an interplay between ligand flexibility and antagonist conformational plasticity. The ActB:Fst288 structure reveals how Fst288 adapts its binding mode to accommodate ligand-specific features, shifting from a cooperative mechanism in the ActA complex to one in which the FSD3 directly engages the ligand to preserve high-affinity antagonism for ActB. These findings refine our understanding of how members of the TGF-β family are regulated by structurally diverse antagonists that have evolved distinct mechanisms to achieve ligand selectivity.

## Experimental procedures

### Protein purification and complex generation

Individual proteins were produced and purified as previously described (8, 11, 12). All the experiments were performed with proteins produced and purified by the authors. Complexes were formed by adding ligands to an excess of Fst288 at a 1:2.5 M ratio, incubated at 4 °C for few hours and purified on a Superdex 200 column (Amersham Biosciences).

### ActB:Fst288 complex crystal structure determination

Purified ActB:Fst288 complex was concentrated to 6.6 mg mL^−1^ in 0.02 M TRIS pH 7.5 and 0.05 M NaCl before it was mixed 1:1 in a hanging drop experiment with a solution containing 0.15 M ammonium sulfate, 0.1 M HEPES and 15% (w/v) PEG 3350. Diffraction experiments were performed at the Argonne National Laboratory Advanced Photon Source 23ID beamline and processed as previously described (11, 12). Phasing was performed by molecular replacement using Phaser (39) and the CCP4 suite (40), using an AlphaFold3 (41) model of a complex containing a human ActB dimer and two Fst288 molecules. Iterative refinement was carried out with Phenix (42) and Coot (43). Coordinates have been deposited in the PDB (PDB ID: 9Z3T).

### Luciferase reporter assay

The luciferase reporter assay using HEK293-(CAGA)_12_ cells (originally derived from RRID:CVCL_0045) and BRITER osteoblast cells was performed as previously published (5, 12, 14). Cells were seeded in 96-well plates and cultured for 24 h. The growth medium was then removed and replaced with serum-free medium + 0.1% bovine serum albumin containing activin ligands at a concentration of 0.62 nM and BMP2 at 2.5 nM, which was mixed with twofold serial dilutions of Fst288. After 18 h, the cells were lysed, and luminescence was measured using a Synergy H1 Hybrid plate reader (BioTek). Activity data were imported into GraphPad Prism 8 and analyzed using nonlinear regression with a variable slope to calculate the IC_50_.

### Heparin affinity analysis

Heparin affinity was determined as previously published (11, 12). Namely, 100 μg of Fst288, either alone or in complex with ActB, ActA, or GDF11, was applied to a 1 ml HiTrap column (Amersham Biosciences) and eluted with a linear gradient of up to 2 M NaCl over 120 column volumes.

### Computational analysis

#### Molecular dynamics (MD) simulations

The resolved structure of the ActB:Fst288 complex, along with previously determined structures of Fst288 complexes with ActA (8), GDF8 (11), and GDF11 (12) were used as starting models for MD simulations. Because of the high similarity between GDF8 and GDF11, most analyses were performed on the GDF8 MD simulations. Missing residues were modeled using Chimera's Modeller plugin (44), and the protonation states of ionizable amino acid residues were estimated by PROPKA 3.1 (45). Complexes were solvated in 10 Å-octahedral boxes, neutralized with corresponding number of counterions, and parametrized using the AMBER ff19SB force field for proteins and the OPC model for water (46, 47). Systems were subjected to geometry optimization in AMBER22 with periodic boundary conditions applied in all directions (48). The optimized systems were gradually heated from 100 to 298 K over 1 ns under constant volume and then equilibrated for 7 ns at a constant pressure, followed by productive, unconstrained MD simulations lasting 300 ns, in triplicate. Starting positions of ActB^A^ (featuring swapped prehelix and wrist helix regions: 335–362 residues in ActB were replaced by 353–380 residues in ActA) and ActA^B^ (featuring swapped prehelix and wrist helix regions: 353–380 residues in ActA were replaced by residues 335–362 in ActB) chimeras were modeled in complex with Fst288 using AlphaFold3 (41). Apo ligand structures were obtained by removing Fst288 from the complexes, whereas the Fst288 dimer was generated by deleting GDF8 from the corresponding complex structure. The same MD simulation protocol described above was applied to all systems. Trajectory processing and data analysis were performed using the CPPTRAJ module implemented in AMBER (49). For enthalpy calculations of ligand:Fst288 5 replicas of 50 ns MD simulations were run. Interaction enthalpies of ligand:Fst288 complexes were computed from 3000 frames taken from the final 40 ns of each simulation using the MM-GBSA protocol and decomposed into *per-residue* contributions (50).

#### Umbrella sampling simulations

Umbrella sampling simulations of ActB:Fst288, ActA:Fst288, and GDF8:Fst288 complexes were performed using GROMACS 2022.4 (51), employing periodic boundary conditions in all directions. The systems were set up in 10.0 nm × 14.0 nm × 25.0 nm rectangular boxes, solvated using the TIP3P water model, and neutralized with the appropriate number of counterions. Each system was oriented so that Fst288 was aligned with the z-axis, along which it was pulled out by a harmonic spring with force constant of 3000 kJ mol^−1^ nm^−2^, moving away from the center of mass of the ligand or ligand:Fst288 complex at constant velocity of 0.01 nm ps^−1^. To prevent the rest of the system from being displaced along with Fst288, a harmonic position restraint with a force constant 5000 kJ mol^−1^ nm^−2^ was imposed on the central region (part of the β-sheets and the wrist helix) of the opposite ligand chain that is not in direct contact with the Fst288 molecule being pulled. For the subsequent windows simulations, snapshots were extracted from the pulling simulations such that the z-component of the ligand-Fst288 or [ligand:Fst288]-Fst288 distance increased by 0.2 nm between neighboring windows, accounting for approximately 65 windows in total. For each window, a 50 ns or 70 ns (in case of [Fst288]_2_:ActB and [Fst288]_2_:GDF8) MD simulation was performed using a harmonic force constant of 2000 kJ mol^−1^ nm^−2^. If necessary, additional windows using a force constant of 3000 kJ mol^−1^ nm^−2^ were added to ensure sufficient overlap between histograms. Potential of mean force (PMF) curves were calculated using the Weighted Histogram Analysis Method (WHAM) with the *gmx wham* tool in GROMACS (52).

## Data availability

The activin B:Fst288 structure has been deposited in PDB under ID: 9Z3T. Software information: AlphaFold3 (https://alphafoldserver.com/), AMBER22 and AmberTools23 (https://ambermd.org/), CCP4 suite (https://www.ccp4.ac.uk/), Coot (https://www2.mrc-lmb.cam.ac.uk/personal/pemsley/coot/), GROMACS 2022.4 (https://www.gromacs.org/), MODELLER (https://salilab.org/modeller/), Phenix (https://www.phenix-online.org/), PROPKA3.1(https://server.poissonboltzmann.org/pdb2pqr), UCSF Chimera 1.17.3 (https://www.cgl.ucsf.edu/chimera/). Data to reproduce the theoretical calculations are available from LH or TBT upon request (hokla@ucmail.uc.edu; Tom.Thompson@uc.edu).

## Supporting information

This article contains supporting information.

## Conflict of interest

The authors declare the following financial interests/personal relationships which may be considered as potential competing interests: EJG is employed by Regeneron Pharmaceuticals.

## References

[1] Hinck A.P., Mueller T.D., Springer T.A. (2016). Structural biology and evolution of the TGF-β family. Cold Spring Harb Perspect. Biol..

[2] Hashimoto O., Kawasaki N., Tsuchida K., Shimasaki S., Hayakawa T., Sugino H. (2000). Difference between follistatin isoforms in the inhibition of activin signalling. Cell Signal..

[3] Sidis Y., Mukherjee A., Keutmann H., Delbaere A., Sadatsuki M., Schneyer A. (2006). Biological activity of follistatin isoforms and Follistatin-Like-3 is dependent on differential cell surface binding and specificity for activin, myostatin, and bone morphogenetic proteins. Endocrinology.

[4] Matzuk M.M., Lu N., Vogel H., Sellheyer K., Roop D.R., Bradley A. (1995). Multiple defects and perinatal death in mice deficient in follistatin. Nature.

[5] Cash J.N., Angerman E.B., Kattamuri C., Nolan K., Zhao H., Sidis Y. (2012). Structure of Myostatin·Follistatin-like 3. J. Biol. Chem..

[6] Schneyer A., Schoen A., Quigg A., Sidis Y. (2003). Differential binding and neutralization of activins A and B by follistatin and follistatin like-3 (FSTL-3/FSRP/FLRG). Endocrinology.

[7] Chang C. (2016). Agonists and antagonists of TGF-β family ligands. Cold Spring Harb Perspect. Biol..

[8] Thompson T.B., Lerch T.F., Cook R.W., Woodruff T.K., Jardetzky T.S. (2005). The structure of the follistatin:activin complex reveals antagonism of both type I and type II receptor binding. Dev. Cell.

[9] Lerch T.F., Shimasaki S., Woodruff T.K., Jardetzky T.S. (2007). Structural and biophysical coupling of heparin and activin binding to follistatin isoform functions. J. Biol. Chem..

[10] Stamler R., Keutmann H.T., Sidis Y., Kattamuri C., Schneyer A., Thompson T.B. (2008). The structure of FSTL3·Activin A complex: differential binding of N-terminal domains influences follistatin-type antagonist specificity. J. Biol. Chem..

[11] Cash J.N., Rejon C.A., McPherron A.C., Bernard D.J., Thompson T.B. (2009). The structure of myostatin:follistatin 288: insights into receptor utilization and heparin binding. EMBO J..

[12] Walker R.G., Czepnik M., Goebel E.J., McCoy J.C., Vujic A., Cho M. (2017). Structural basis for potency differences between GDF8 and GDF11. BMC Biol..

[13] Keutmann H.T., Schneyer A.L., Sidis Y. (2004). The role of follistatin domains in follistatin biological action. Mol. Endocrinol..

[14] Cash J.N., Angerman E.B., Keutmann H.T., Thompson T.B. (2012). Characterization of follistatin-type domains and their contribution to myostatin and activin A antagonism. Mol. Endocrinol..

[15] Harrington A.E., Morris-Triggs S.A., Ruotolo B.T., Robinson C.V., Ohnuma S., Hyvönen M. (2006). Structural basis for the inhibition of activin signalling by follistatin. EMBO J..

[16] Schneyer A.L., Sidis Y., Gulati A., Sun J.L., Keutmann H., Krasney P.A. (2008). Differential antagonism of activin, myostatin and growth and differentiation factor 11 by wild-type and mutant follistatin. Endocrinology.

[17] Harrison C.A., Chan K.L., Robertson D.M. (2006). Activin-A binds follistatin and type II receptors through overlapping binding sites: generation of mutants with isolated binding activities. Endocrinology.

[18] Sidis Y., Schneyer A.L., Sluss P.M., Johnson L.N., Keutmann H.T. (2001). Follistatin: essential role for the N-terminal domain in activin binding and neutralization. J. Biol. Chem..

[19] Nakatani M., Takehara Y., Sugino H., Matsumoto M., Hashimoto O., Hasegawa Y. (2008). Transgenic expression of a myostatin inhibitor derived from follistatin increases skeletal muscle mass and ameliorates dystrophic pathology in mdx mice. FASEB J..

[20] Thompson T.B., Woodruff T.K., Jardetzky T.S. (2003). Structures of an ActRIIB:activin A complex reveal a novel binding mode for TGF-β ligand:receptor interactions. EMBO J..

[21] Wang X., Fischer G., Hyvönen M. (2016). Structure and activation of pro-activin A. Nat. Commun..

[22] Chu K.-Y., Malik A., Thamilselvan V., Martinez-Hackert E. (2022). Type II BMP and activin receptors BMPR2 and ACVR2A share a conserved mode of growth factor recognition. J. Biol. Chem..

[23] Cotton T.R., Fischer G., Wang X., McCoy J.C., Czepnik M., Thompson T.B. (2018). Structure of the human myostatin precursor and determinants of growth factor latency. EMBO J..

[24] Padyana A.K., Vaidialingam B., Hayes D.B., Gupta P., Franti M., Farrow N.A. (2016). Crystal structure of human GDF11. Acta Cryst. F.

[25] Goebel E.J., Kattamuri C., Gipson G.R., Krishnan L., Chavez M., Czepnik M. (2022). Structures of activin ligand traps using natural sets of type I and type II TGFβ receptors. iScience.

[26] Goebel E.J., Corpina R.A., Hinck C.S., Czepnik M., Castonguay R., Grenha R. (2019). Structural characterization of an activin class ternary receptor complex reveals a third paradigm for receptor specificity. Proc. Natl. Acad. Sci. U. S. A..

[27] Namwanje M., Brown C.W. (2016). Activins and inhibins: roles in development, physiology, and disease. Cold Spring Harb Perspect. Biol..

[28] Wijayarathna R., de Kretser D.M. (2016). Activins in reproductive biology and beyond. Hum. Reprod. Update.

[29] Fang D.Y.P., Lu B., Hayward S., de Kretser D.M., Cowan P.J., Dwyer K.M. (2016). The role of activin A and B and the benefit of follistatin treatment in renal ischemia-reperfusion injury in mice. Transpl. Direct.

[30] Krepinsky J.C. (2022). Activin B, a new player in kidney fibrosis?. J. Pathol..

[31] Nagayama I., Takei Y., Takahashi S., Okada M., Maeshima A. (2025). The activin-follistatin system: key regulator of kidney development, regeneration, inflammation, and fibrosis. Cytokine Growth Factor Rev..

[32] Thompson T.B., Cook R.W., Chapman S.C., Jardetzky T.S., Woodruff T.K. (2004). Beta A versus beta B: is it merely a matter of expression?. Mol. Cell Endocrinol.

[33] Nolan K., Kattamuri C., Rankin S.A., Read R.J., Zorn A.M., Thompson T.B. (2016). Structure of Gremlin-2 in complex with GDF5 gives Insight into DAN-Family-Mediated BMP antagonism. Cell Rep..

[34] Mast E.M., Leach E.A.E., Thompson T.B. (2024). Characterization of erythroferrone oligomerization and its impact on BMP antagonism. J. Biol. Chem..

[35] Walker R.G., Kattamuri C., Goebel E.J., Zhang F., Hammel M., Tainer J.A. (2021). Heparin-mediated dimerization of follistatin. Exp. Biol. Med. (Maywood).

[36] Zhang F., Beaudet J.M., Luedeke D.M., Walker R.G., Thompson T.B., Linhardt R.J. (2012). Analysis of the Interaction between Heparin and Follistatin and Heparin and Follistatin Ligand Complexes Using Surface Plasmon Resonance. Biochemistry.

[37] Goebel E.J., Ongaro L., Kappes E.C., Vestal K., Belcheva E., Castonguay R. (2022). The orphan ligand, activin C, signals through activin receptor-like kinase 7. Elife.

[38] Vestal K.A., Kattamuri C., Koyiloth M., Ongaro L., Howard J.A., Deaton A.M. (2024). Activin E is a transforming growth factor β ligand that signals specifically through activin receptor-like kinase 7. Biochem. J..

[39] McCoy A.J., Grosse-Kunstleve R.W., Adams P.D., Winn M.D., Storoni L.C., Read R.J. (2007). Phaser crystallographic software. J. Appl. Crystallogr..

[40] Agirre J., Atanasova M., Bagdonas H., Ballard C.B., Baslé A., Beilsten-Edmands J. (2023). The CCP4 suite: integrative software for macromolecular crystallography. Acta Cryst. D.

[41] Abramson J., Adler J., Dunger J., Evans R., Green T., Pritzel A. (2024). Accurate structure prediction of biomolecular interactions with AlphaFold 3. Nature.

[42] Liebschner D., Afonine P.V., Baker M.L., Bunkóczi G., Chen V.B., Croll T.I. (2019). Macromolecular structure determination using X-rays, neutrons and electrons: recent developments in phenix. Acta Crystallogr. D Struct. Biol..

[43] Emsley P., Lohkamp B., Scott W.G., Cowtan K. (2010). Features and development of coot. Acta Crystallogr. D Biol. Crystallogr..

[44] Šali A., Blundell T.L. (1993). Comparative protein modelling by satisfaction of spatial restraints. J. Mol. Biol..

[45] Olsson M.H.M., Søndergaard C.R., Rostkowski M., Jensen J.H. (2011). PROPKA3: consistent treatment of internal and surface residues in empirical pKa predictions. J. Chem. Theor. Comput..

[46] Tian C., Kasavajhala K., Belfon K.A.A., Raguette L., Huang H., Migues A.N. (2020). ff19SB: Amino-acid-specific protein backbone parameters trained against quantum mechanics energy surfaces in solution. J. Chem. Theor. Comput..

[47] Izadi S., Anandakrishnan R., Onufriev A.V. (2014). Building water models: a different approach. J. Phys. Chem. Lett..

[48] Salomon-Ferrer R., Case D.A., Walker R.C. (2013). An overview of the Amber biomolecular simulation package. WIREs Comput Mol Sci.

[49] Roe D.R., Cheatham T.E.I. (2013). PTRAJ and CPPTRAJ: software for processing and analysis of molecular dynamics trajectory data. J. Chem. Theor. Comput..

[50] Genheden S., Ryde U. (2015). The MM/PBSA and MM/GBSA methods to estimate ligand-binding affinities. Expert Opin. Drug Discov..

[51] Abraham M.J., Murtola T., Schulz R., Páll S., Smith J.C., Hess B. (2015). GROMACS: high performance molecular simulations through multi-level parallelism from laptops to supercomputers. SoftwareX.

[52] Hub J.S., de Groot B.L., van der Spoel D. (2010). g_wham—A free weighted histogram analysis implementation including robust error and autocorrelation estimates. J. Chem. Theor. Comput..

