# Supplementary material for: The crystal structure of the activin B:Fst288 complex and computational insights into the broad antagonistic activity and specificity of follistatin
Source: J Biol Chem. 2026 Aug 17;302(10):113459. doi: 10.1016/j.jbc.2026.113459 (PMC13590098; doi:10.1016/j.jbc.2026.113459)
Supplement: Supplementary file 1 [file mmc1.docx]

**Supporting Information**

**The crystal structure of the activin B:Fst288 complex and computational insights into the broad antagonistic activity and specificity of follistatin**

Lucija Hok^1,2^, Ryan G. Walker^1^, James A. Howard^3^, Melissa M. Gouge^1^, Eleanor M. Mast^1^, Chandramohan Kattamuri^1^, Erich J. Goebel^1,4^, Thomas B. Thompson^1^

^1^Department of Molecular & Cellular Biosciences, College of Medicine, University of Cincinnati, Cincinnati, OH 45267, USA

^2^Institute of Medical Physics and Biophysics, Universitätsklinikum Münster, Center for Soft Nanoscience, Münster, Germany (Current affiliation)

^3^Department of Pharmacology & Systems Physiology, College of Medicine, University of Cincinnati, Cincinnati, OH 45267, USA

^4^Regeneron Pharmaceuticals, Tarrytown, NY 10591, USA (Current affiliation)

*Corresponding author. E-mail: Tom.Thompson@uc.edu

**Table of Contents**

| **Content** | **Pages** |
| --- | --- |
| Supplementary figures S1−S13 | S2–S14 |
| Supplementary table S1 | S15 |

**Figure S1 |** **Domain Organization of Fst288.** A) Sequence of four Fst288 domains with the secondary structure shown above. Heparin binding sequence (HBS) in FSD1 is given in cyan. B) Structures of individual Fst288 domains from the ActB:Fst288 complex (PDB ID: 9Z3T) aligned through the Kazal1 helix, with connecting disulfide bonds indicated.

**Figure S2 | Inhibitory activity of Fst288 against BMP2 as a negative control.** Luciferase reporter assays showing the inhibitory activity of increasing concentrations of Fst288 against a constant concentration of 2.5 nM BMP2 in BRITER osteoblast cells. The curve represents the mean of three independent experiments, with each assay performed in triplicate.

**Figure S3 |** **Structural Comparison of Individual Fst288 Domains in Complexes with Activins.** Position of the Fst domains in the A) ActA and B) GDF8 complexes relative to ActB aligned through ligand chain_1_. Fst domains are colored purple in the ActB complex, magenta in ActA, and blue in GDF8. Differences in the centers of mass of individual domains relative to those in the ActB complex are given in Å. Only the ActB ligand is shown, in the beige surface representation.

**Figure S4 |** **Analysis of Ligand Flexibility in Apo and Complex MD Simulations.** RMSD graphs for backbone atoms of ligands in MDS of their A) apo forms and B) complexes with Fst288. Principal Component Analysis (PCA) showing the distribution of ligand conformations throughout MDS of their C) apo forms and D) complexes with Fst288, shown as density plots of the first two principal components. The density of points is indicated by a color gradient: yellow represents higher density, and dark blue represents lower density. Positions of the prehelix and wrist helix of ligands during MDS of their E) apo forms and F) complexes with Fst288, together with the average structures for reference. For clarity, the occupied volume of only one chain is shown (in blue), however, the calculated values represent averages from both chains. Interchain angle in MDS of G) apo forms and H) complexes measured between V392_1_-C372_1_-V392_2_ in ActB, I410_1_-C390_1_-I410_2_ in ActA, and I359_1_-C339_1_-I359_2_ in GDF8. I) Superposition of the starting and final frames of the apo MDS for ActB (left), ActA (middle), and GDF8 (right). On the left, ActB from the ActRIIB complex (PDB: 7U5O) is shown in light green. Ligands are aligned through one chain (not shown) to illustrate changes in the interchain angle.

**Figure S5 |** **Analysis of Chimeric Ligands Flexibility in Apo and Complex MD Simulations.** Confidence metrics for AF3 models of A) ActB^A^ and B) ActA^B^ in complex with Fst288. RMSD graphs for backbone atoms of ActB^A^ and ActA^B^ mutants in MDS of their C) apo forms and D) complexes with Fst288. Principal Component Analysis (PCA) results for ActB^A^ and ActA^B^ mutants from MDS of their E) apo forms and F) complexes with Fst288, shown as density plots of the first two principal components. The density of points is indicated by a color gradient: yellow represents higher density, and dark blue represents lower density. Positions of the prehelix and wrist helix regions of ActB^A^ and ActA^B^ mutants during MDS of their G) apo forms and H) complexes with Fst288, together with the average structures for reference. For clarity, the occupied volume of only one chain is shown (in blue), however, the calculated values represent averages from both chains. Interchain angle in MDS of I) apo forms and J) complexes measured between V392_1_-C372_1_-V392_2_ in ActB^A^, and I410_1_-C390_1_-I410_2_ in ActA^B^.

**Figure S6 | Analysis of Fst288 Flexibility in MD Simulations of the Activin Complexes.** RMSD graphs for backbone atoms of Fst288 domains in complexes with A) ActB, B) ActA, and C) GDF8. D) Left: Position of FSD3 relative to ActB at the beginning (blue) and end (red) in MD simulation of the AcB:Fst288 complex. Right: Distance graphs between Fst288[R266-CZ] and ActB[E388-CD] on both chains over the course of the simulation.

**Figure S7 | Analysis of Fst288 Flexibility in Apo MD Simulations.** RMSD graphs for backbone atoms of Fst288 in A) single-molecule MD simulation and B) MD simulation of a complex of two Fst288 molecules, both in the absence of ligands. C) On the left: final frame of the apo MD simulation of a single Fst288 molecule showing the interaction between positively charged residues in the heparin binding sequence (HBS) of FSD1 and negatively charged residues in FSD3. FSD1 is colored light brown with the HBS in light blue, and FSD3 is shown in dark blue. On the right: electrostatic potential surface of a single Fst288 molecule after 300 ns calculated using Adaptive Poisson-Boltzmann Solver (APBS), with positive regions in blue and negative regions in red. D) On the left: final frame of the apo MD simulation of two Fst288 molecules showing the interaction between positively charged residues in the HBS of FSD1 from one molecule and negatively charged residues in FSD3 of the other. The ND is colored red, FSD1 is light brown with HBS in light blue, and FSD3 is dark blue. On the right: electrostatic potential surface of two Fst288 molecules after 350 ns calculated using APBS, with positive regions in blue and negative regions in red.

**Figure S8 | Type I and Fst[1]-Fst[2] Interfaces in GDF8 and GDF11 Complexes.** A) Top: The buried surface area on chain_1_ by the ND that is shared between ActB and GDF8 is colored red. ActB chain_1_ is shown in beige and chain_2_ in white surface representation. Middle and bottom: Detailed view of the ND helix binding site within the concave region of GDF8 and GDF11. B) Top: The buried surface area on chain_2_ by the ND that is shared between ActB and GDF8 is colored pink, while the area specific to GDF8 is shown in blue and to ActB in purple. Middle and bottom: Detailed view of the interaction between the ND loops and the N-terminus and wrist helix of GDF8 and GDF11. C) Top: The ND and FSD3 domains from the ActB and GDF8 complexes. Middle and bottom: Detailed view of the interactions between FSD3[2], ND[1] and the ligand in the GDF8 and GDF11 complexes.

**Figure S9 | Type I Interface of Chimeric Ligands with the ND Domain.** Superposition of the final frames from MD simulations of Fst288 complexes with ActB and ActA mutants (with swapped prehelix and wrist helix regions) onto the crystal structure of ActB:Fst288. Alignment was performed using chain_1_, and only chain_1_ from the crystal structure is shown. The ND helix and chain_2_ from the ActB crystal structure are colored yellow, from the ActB^A^ mutant in purple, and from the ActA^B^ mutant in magenta. Calculated buried surface area values (≥15 Å²) for ND interactions with ActB^A^ and ActA^B^ chains are given in the table below.

**Figure S10 | Umbrella sampling simulations of Fst288:ligand complexes.** A) Histograms, depicting ActB (top), ActA (middle), and GDF8 (bottom), show the probability distribution of the distance between the centers of mass of ligands and Fst288 along the reaction coordinate for each window of umbrella sampling simulations. B) Convergence analysis of the umbrella sampling simulations for ActB (left), ActA (middle) and GDF8 (right).

**Figure S11 | Umbrella sampling simulations of [Fst288]_2_:ligand complexes.** A) Histograms depicting ActB (top), ActA (middle), and GDF8 (bottom) complexes with one Fst288 molecule show the probability distribution of the distance between the centers of mass of the Fst288:ligand complex and second Fst288 along the reaction coordinate for each window of umbrella sampling simulations. B) Convergence analysis of the umbrella sampling simulations for ActB (left), ActA (middle) and GDF8 (right) complexes.

**Figure S12 |** **Computational Evaluation of Gibbs Free Energy between Activins and the ND Domain.** Potential of mean force curves along the reaction coordinate, calculated from umbrella sampling simulations for dissociation of the ND from ligands, with corresponding Δ*G* values indicated on right (in kcal mol^−1^). A 20 ns MD simulation was performed for each window.

**Figure S13 | Insight into the Origin of Fst288 Specificity through Sequence Alignment of TGF-β Ligands.** A) Sequence alignment of the strongly antagonized activins, moderately antagonized BMPs, and non-binding TGF-β ligands. Conserved amino acids among all ligands are highlighted in blue, residues that differ between antagonized and non-binding ligands are shown in red, and those that differ between the strongly and moderately antagonized ligands are shown in orange. The secondary structure of ActB is shown at the top of the alignment. B) On the left, the red and orange residues, and on the right, the blue residues from the sequence alignment are mapped onto the ActB structure in both side (left) and top (right) views.

**Table S1 |** A) Calculated MM-GBSA enthalpy between the two Fst288 molecules and ligands with values from five MD replicas. B) Distribution of calculated MM-GBSA enthalpy across individual Fst288 domains with values of both domains from five MD replicas. C) Calculated MM-GBSA enthalpy between the two Fst288 molecules with values from five MD replicas.

| **A)** | | | | | | | |
| --- | --- | --- | --- | --- | --- | --- | --- |
| **Δ*H*{[Fst288]_2_:ligand} (kcal mol^−1^)** | | | | | | | |
|  | #1 | #2 | #3 | #4 | #5 | AVG | STD |
| **ActB** | −191.7 | −206.4 | −202.3 | −219.3 | −194.1 | **−202.7** | *9.8* |
| **ActA** | −108.7 | −112.9 | −119.2 | −120.0 | −105.6 | **−113.3** | *5.7* |
| **GDF8** | −118.6 | −128.9 | −121.9 | −122.3 | −119.2 | **−122.2** | *3.7* |
| **B)** | | | | | | | |
| **Distribution of Δ*H*{[Fst288]_2_:ActB} across Fst288 domains (kcal mol^−1^)** | | | | | | | |
|  | #1 | #2 | #3 | #4 | #5 | AVG | STD |
| **ND** | −28.4 | −26.9 | −33.9 | −31.9 | −28.4 | **−30.5** | *2.6* |
|  | −31.3 | −32.0 | −26.7 | −34.5 | −31.5 |  |  |
| **FSD1** | 0.5 | 0.1 | 5.3 | −0.5 | −0.7 | **2.4** | *2.5* |
|  | 4.6 | 2.0 | 5.7 | 1.7 | 5.6 |  |  |
| **FSD2** | −26.0 | −29.0 | −28.5 | −25.7 | −21.9 | **−26.1** | *2.2* |
|  | −25.0 | −23.9 | −28.6 | −27.6 | −25.3 |  |  |
| **FSD3** | −0.8 | 0.2 | 1.8 | 0.6 | 1.7 | **0.7** | *1.8* |
|  | 1.6 | 1.5 | −4.1 | 2.4 | 1.9 |  |  |
| **Distribution of Δ*H*{[Fst288]_2_:ActA} across Fst288 domains (kcal mol^−1^)** | | | | | | | |
|  | #1 | #2 | #3 | #4 | #5 | AVG | STD |
| **ND** | −12.9 | −16.3 | −18.7 | −10.0 | −12.2 | **−17.5** | *4.4* |
|  | −21.4 | −17.5 | −22.8 | −20.0 | −23.2 |  |  |
| **FSD1** | 3.5 | 3.4 | 3.7 | 4.7 | 3.9 | **3.4** | *1.7* |
|  | 4.2 | 4.0 | −1.5 | 3.9 | 4.2 |  |  |
| **FSD2** | −19.1 | −21.6 | −16.7 | −18.2 | −18.9 | **−18.0** | *2.8* |
|  | −19.9 | −13.4 | −12.7 | −19.1 | −20.3 |  |  |
| **FSD3** | 3.5 | 4.2 | 3.4 | 4.0 | 3.9 | **3.4** | *0.5* |
|  | 2.6 | 3.0 | 2.7 | 3.3 | 3.1 |  |  |
| **Distribution of Δ*H*{[Fst288]_2_:GDF8} across Fst288 domains (kcal mol^−1^)** | | | | | | | |
|  | #1 | #2 | #3 | #4 | #5 | AVG | STD |
| **ND** | −21.0 | −21.7 | −19.3 | −20.3 | −17.2 | **−20.2** | *1.5* |
|  | −22.3 | −21.2 | −19.5 | −18.5 | −20.9 |  |  |
| **FSD1** | 3.7 | 5.7 | 8.8 | 6.1 | 7.0 | **5.2** | *2.0* |
|  | 4.2 | 2.8 | 6.5 | 2.1 | 4.6 |  |  |
| **FSD2** | −22.9 | −20.3 | −28.3 | −14.2 | −21.0 | **−21.6** | *3.4* |
|  | −19.5 | −23.1 | −20.3 | −23.7 | −22.5 |  |  |
| **FSD3** | 5.0 | 4.3 | 4.1 | 3.9 | 4.9 | **4.6** | *0.5* |
|  | 5.0 | 5.5 | 4.3 | 4.2 | 5.2 |  |  |
| **C)** | | | | | | | |
| **Δ*H*{Fst288[1]:Fst288[2]} (kcal mol^−1^)** | | | | | | | |
|  | #1 | #2 | #3 | #4 | #5 | AVG | STD |
| **ActB** | 10.1 | 12.3 | −0.5 | 2.5 | 1.2 | **5.1** | *5.1* |
| **ActA** | −41.4 | −40.6 | −37.4 | −46.0 | −44.5 | **−42.0** | *3.0* |
| **GDF8** | −38.0 | −34.4 | −32.5 | −33.3 | −31.6 | **−34.0** | *2.2* |
